# Substitution-induced spin-splitted surface states in topological insulator (Bi_1−x_Sb_x_)_2_Te_3_

**DOI:** 10.1038/srep08830

**Published:** 2015-03-06

**Authors:** Xiaoyue He, Hui Li, Lan Chen, Kehui Wu

**Affiliations:** 1 Institute of Physics, Chinese Academy of Science, Beijing 100190, China; 2 Key Laboratory of Standardization and Measurement for Nanotechnology, Chinese Academy of Sciences, National Center for Nanoscience and Technology, Beijing 100190, China; 3 Collaborative Innovation Center of Quantum Matter, Beijing 100871, China

## Abstract

We present a study on surface states of topological insulator (Bi_1−x_Sb_x_)_2_Te_3_ by imaging quasiparticle interference patterns (QPI) using low temperature scanning tunneling microscope. Besides the topological Dirac state, we observed another surface state with chiral spin texture within the conduction band range. The quasiparticle scattering in this state is selectively suppressed. Combined with first-principles calculations, we attribute this state to a spin-splitted band induced by the substitution of Bi with Sb atoms. Our results demonstrate that the coexistence of topological order and alloying may open wider tunability in quantum materials.

Three dimensional (3D) topological insulator (TIs) is a new state of quantum matter with nontrivial gapless surface states in the bulk energy gap where the directions of spin and moment are locked. The topological surface state (TSS) is originated from strong spin-orbit coupling (SOC) effect, and robust to nonmagnetic perturbation, as the topological order is protected by the particle number conservation and time-reversal (TR) symmetry^1^,^2^,^3^. Such unique electronic properties provide a new ground not only for novel quantum phenomena (such as magnetic monopole and Majorana fermions) but also for applications in spintronics and quantum computing^4^,^5^,^6^,^7^.

Among all 3D TIs, bismuth telluride (Bi_2_Te_3_) has been widely investigated due to its large SOC gap. However, the position of Dirac point (DP) for pure Bi_2_Te_3_ is beneath its bulk valence bands maxima, making it difficult for application in electronic devices. Furthermore, although significant efforts have been made to realize the surface transport, the progress is still hampered by large unintentional bulk contribution to the total conductance caused by Te vacancies. Recently, improved bulk-insulating property was achieved in the Bi-based ternary (Bi_1−x_Sb_x_)_2_Te_3_ (BST)^8^,^9^,^10^,^11^, which has a tetradymite structure similar to Bi_2_Te_3_. The existence of TSS in BST was confirmed by angle resolved photoemission spectroscopy (ARPES) and electronic transport measurements for 0 ≤ x ≤ 1. The energy position of DP for BST relative to the Fermi level can be tuned by adjusting the stoichiometry of the Bi and Sb, eventually leading to be an ideal TI with insulating bulk. Such great advantage has been substantially demonstrated by the recent discovery of quantum anomalous Hall Effect (QAHE) in Cr-doped BST films^12^. However, despite the application in transport studies, fundamental properties of BST have not been fully understood yet. For example, regarding the random alloying of Bi and Sb atoms in BST, the effects of structural disorder and on the electronic structure and transport properties are still unknown.

In this article, besides the conventional TSS, we found in ternary compound (Bi_1−x_Sb_x_)_2_Te_3_ films a new surface state located within the conductance band. This band is absent in binary compounds Bi_2_Te_3_. Remarkbly, the band also exhibits chiral spin texture, as revealed by quasiparticle interference (QPI) patterns. First principle calculations suggest that this band is originated from the Sb/Bi layer due to the random substitution of Bi by Sb atoms. This demonstrates that alloying of semiconductor components provides not only a method to tune the semiconducting gap or the existing surface state, but also capable of creating new exotic electronic states for potential applications.

## Results

### Characterization of (Bi_1−x_Sb_x_)_2_Te_3_ surface

**Figure 1:**
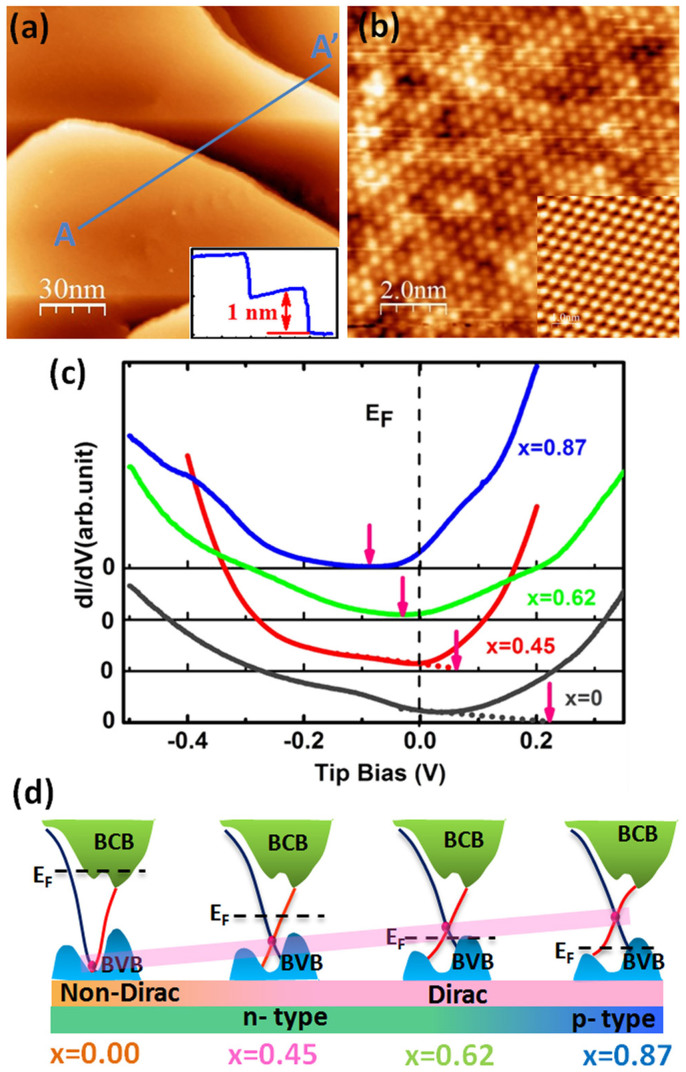
STM images and differential conductance spectroscopy. (a) The STM image (150 nm × 150 nm) of (Bi_1−x_Sb_x_)_2_Te_3_ film with x = 0.45. Scanning conditions: V = −1.72 V, I = 207 pA. The inset shows line profile along the blue line marked AA′ in (a). (b) The atomic-resolution STM image (10 nm × 10 nm) obtained on terrace in (a). Scanning parameter is 0.298 V and 207 pA. The lattice constant is about 0.44 nm. The inset shows the atomic-resolution STM image of the Bi_2_Te_3_ films (5 nm × 5 nm, −0.3 V, 270 pA). (c) Typical dI/dV curves obtained on (Bi_1−x_Sb_x_)_2_Te_3_ films with x = 0 (black line), 0.45 (red line), 0.62 (green line) and 0.87 (blue line), respectively. The pink arrows show the approximate positions of Dirac point (E_D_). (d) Schematic illustration of band structure and surface state of (Bi_1−x_Sb_x_)_2_Te_3_ films varied with x value. The pink bar shows the evolution of position of Dirac Point with different x values.

The morphology of the atomic flat terraces on the surface of a (Bi_0.45_Sb_0.55_)_2_Te_3_ film is illustrated in Fig. 1(a). The single step height is about 1 nm, corresponding to one quintuple layer. High-resolution STM image (Fig. 1(b)) indicates a well-ordered hexagonal close-packed structure with a lattice constant of 4.4 ± 0.1 Å, which is close to that of Bi_2_Te_3_ (inset in Fig. 1(b)). But the brightness of atoms is not identical, which is different from the Bi_2_Te_3_. Considering that STM image contains the contribution from local density of states (LDOS), the different brightness may be the result of electronic distortions caused by random substitution of Bi with Sb. It is similar to the surfaces of Bi_1−x_Sb_x_ alloy^13^, Bi_2+x_Te_x_Se^14^ and Bi_1.5_Sb_0.5_Te_1.7_Se_1.3_^15^. The STS measurements were performed to investigate the electronic structures of BST. We obtained the typical dI/dV curves of (Bi_1−x_Sb_x_)_2_Te_3_ films with different Sb composition shown in Fig. 1(c). The positions of DP, corresponding to the minima of LDOS or the extension of the linearly dispersing Dirac bands to bias axis^16^,^17^, are about −230 meV, −50 meV, 15 meV and 98 meV for x = 0, 0.45, 0.62, and 0.87, respectively. Obviously, the energy of DP gradually lifts up from the valence band as x increases, as illustrated in the schematic band diagram (Fig. 1(d)). From it, we estimate that an intrinsic topological insulator, where the DP is in the bulk energy gap and close to the Fermi level, will be achieved in sample with x = 0.5 ± 0.1, in agree with transport results in the (Bi_1−x_Sb_x_)_2_Te_3_ films^10^.

### Surface states measurement from quasiparticle interference (QPI) analysis

**Figure 2:**
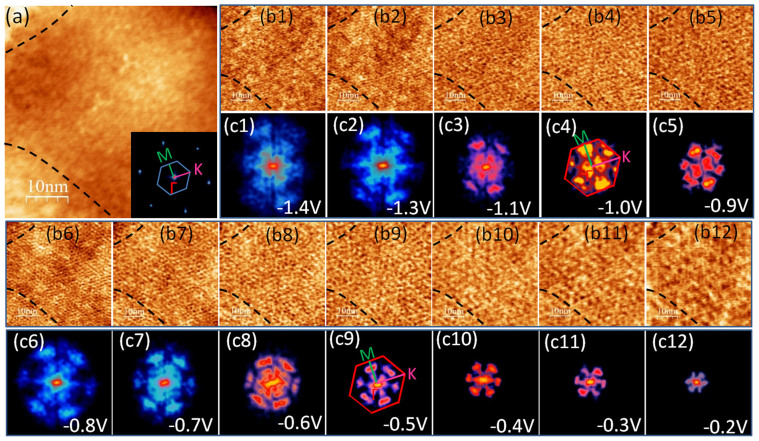
QPI patterns on the (Bi_0.45_Sb_0.55_)_2_Te_3_ films surface. (a) STM image of the (Bi_0.44_Sb_56_)_2_Te_3_ surface (50 nm × 50 nm). The inset shows the FFT result of (a), and the first Brillouin Zone is added. (b1–b12) dI/dV maps of the same area as (a) at different tip bias. The values of tip bias are shown in figures. The scanning current was 380 pA. Each map has 256 × 256 pixels. The dash lines in (a) and (b1–b12) are marks to indicate the relative positions in STM images. (c1–c12) FFT results of the dI/dV maps (b1–b12) respectively. The first Brillouin Zone of (Bi_0.44_Sb_56_)_2_Te_3_ surface is superimposed in (C4) and (C9) to show the directions of quasipartilce scattering.

To obtain how the surface states disperse in momentum space, we need to investigate the standing waves caused by quasiparticle interference (QPI) on surface. A series of dI/dV maps of a (Bi_0.45_Sb_0.55_)_2_Te_3_ thin film taken at a tip bias voltage (V_T_) from −0.2 V to −1.5 V are shown in Fig. 2(b1–b12), which exhibit pronounced standing waves throughout the surface. The fast Fourier transformation (FFT) of the dI/dV maps, shown in Fig. 2(c1–c12), indicates the distribution of scattering wave vectors **q** in momentum space. We found there are six-fold symmetric spots with intensity centered along the ΓM direction of the first Brillouin zone at outer edge of FFT patterns. The spots are small at V_T_ between −0.3 and −0.6 V, and get gradually broader when V_T_ is decreasing to more negative side. Next, between −0.9 V and −1.1 V, the QPI patterns dramatically shrink and become intensive again, and then they are gradually broadened and obscured below −1.2 V and vanished until −1.5 V once more.

**Figure 3:**
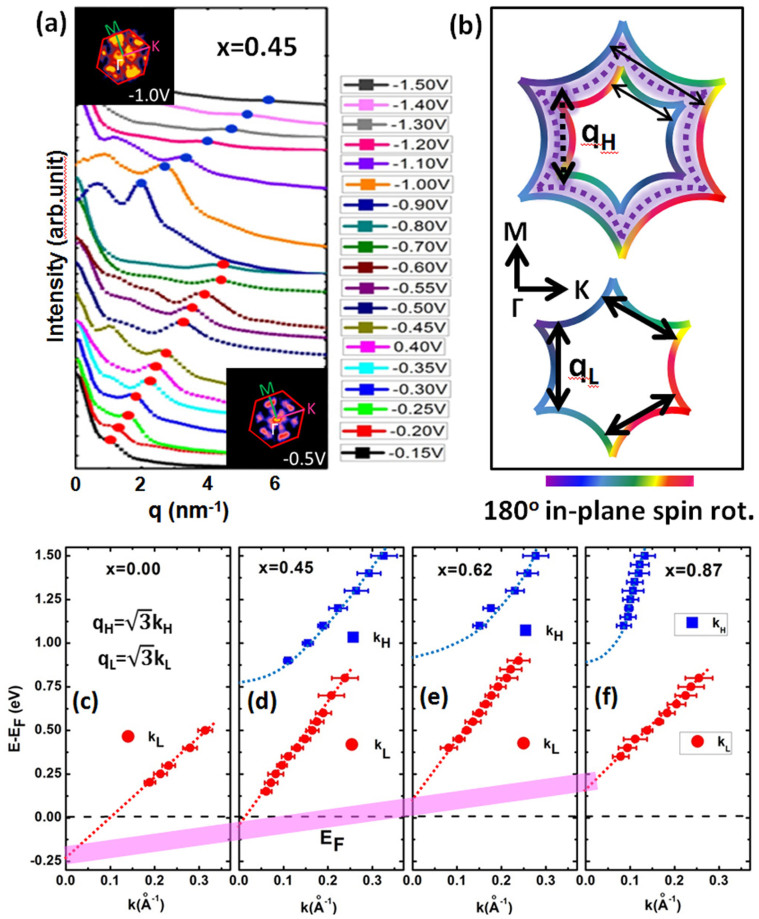
Energy-Momentum dispersion of surface states. (a) The radial integral FFT intensity as a function of distance from Γ point in FFT images with different bias voltages. The blue and red dots mark the peaks corresponding to positions of bright spots in FFT images. The insets show the typical FFT images at high (on the left top) and low energy region (on the right bottom), respectively. (b) Schematics of constant energy contours (CEC) in k space at high and low energy regions. The dash lines in CEC at high energy regions are the average value of inner and outer parts of CEC. The arrows represent the possible quasiparticle scattering channels (q_H_ and q_L_). (c)–(f) Energy-momentum (E–k) dispersion of surface states in (Bi_1−x_Sb_x_)_2_Te_3_ films with different x values. The solid circles and squares represent momentums of the states at low and high energy regions, respectively. The momentum k is determined by the q = √3 k, in which the q values and error bars are determined from the peak positions and FWHM in (a). The pink bar shows the evolution of position of Dirac Point with different x values.

The six-fold symmetric spots in FFT images are related to robust scattering vectors. To better understand the evolution of scattering vectors with energy, we integrate the FFT intensity along the radical direction, and plot it as a function of distance from Γ point, as shown in Fig. 3(a). The peaks marked by dots correspond to the positions of the spots. It is obviously that there are two dispersive bands of scattering vectors: one at the low energy region (from −0.8 V to −0.2 V), the other at high energy region (from −1.5 V to −0.9 V). Similar features have also been observed on the BST surface for x = 0.62 and 0.87. However, in the case of pure Bi_2_Te_3_ (BST for x = 0) only that at the low energy region (−0.5 V < V_T_ < −0.2 V) is observed.

The two different bands of scattering vectors relate to quaiparticle scattering in two different states. The low energy state at 0.15–0.8 eV above E_F_ should be attributed to the topological Dirac surface state. The hexagonally warped Dirac cone (DC) with chiral spin texture in BST^8^ and the protection by time-reversal symmetry result in scattering only in the ΓM direction (the schematic illustration shown in Fig. 3(b) (bottom)), corresponding to six-fold symmetric spots in reciprocal space^18^,^19^. The similar scattering vector along ΓM direction has been reported on Bi_1−x_Sb_x_ alloy^13^, Bi_2_Te_3_^18^, and Bi_2_Se_3_^16^. In constant energy contours (CEC) of DC, the wave vector k along the ΓK direction and the scattering vector q should obey the equation: q = √3 k^18^. Thus, the dispersions of the Dirac states along the ΓK direction can be obtained, as shown in Fig. 3(c)–(f). The linear fittings to the data are perfect, and the slopes provide measurement of the Fermi velocity V_F_, which are 3.58 × 10^−5^, 5.34 × 0^−5^, 5.23 × 10^−5^, and 3.69 × 10^−5^ m/s, corresponding to x = 0, 0.45, 0.62, and 0.87, respectively. In addition, the energy positions of DP for BST films are about −248, −55, 110, and 164 meV, respectively, by estimating the interceptions of the dispersion curves with the energy axis. There is a small deviation of the energy position of DP from measurements in dI/dV curves. The discrepancy may be due to the electrostatic induction by the electric field between tip and sample^20^.

For the high energy state at 0.9–1.5 eV above EF, which is located in the bulk conduction band region, there are also six-fold symmetric spots along the ΓM direction in FFT images, similar as the Dirac state. This suggests backscattering suppression of quasiparticles from a new surface state with chiral spin texture. One possible origination of this state is two dimensional electron gas (2DEG) with Rashba-type splitting on TIs surface, caused by the band bending due to foreign gas/atom adsorption^21^,^22^,^23^. However, although this state may have the same spin chiral texture with the TSS, its energy should be near the bottom of bulk conduction band, around 200–300 meV above EF. In contrast, the new state found in our experiments distributes at higher energy region (0.9 eV to 1.5 eV). Secondly, this state is absent in pure Bi_2_Te_3_ films in our experiment, indicating that it should be related to the substitution of Bi by Sb. Therefore, the possibility of 2DEG as origination of this state is ruled out.

### Assignment of the surface states based on the first-principles calculation

**Figure 4:**
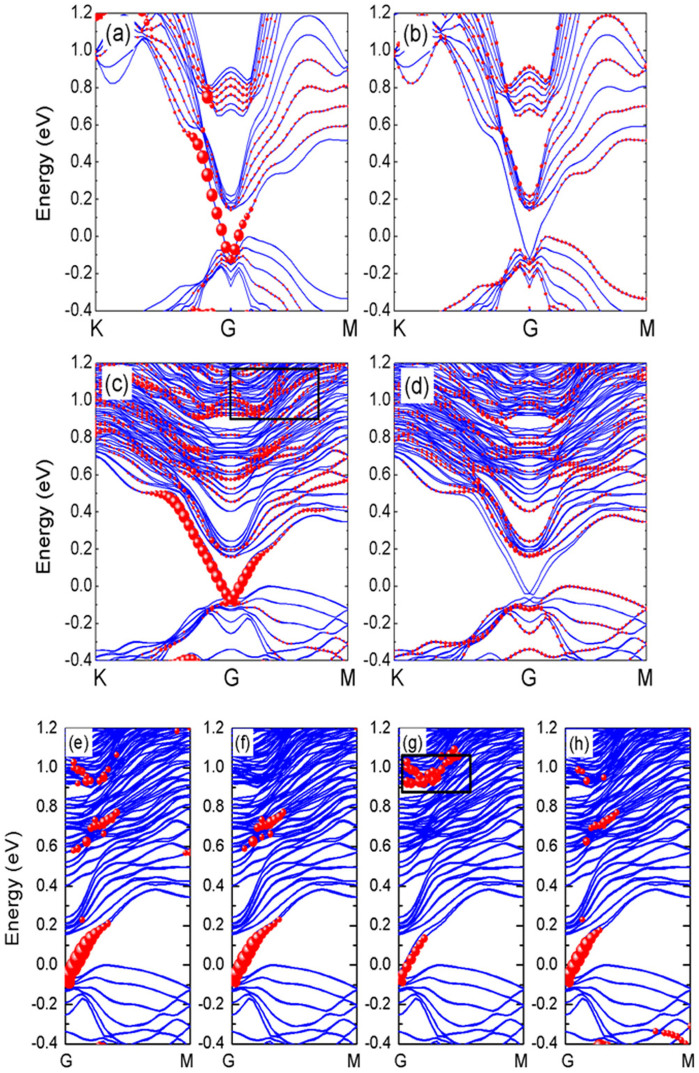
The calculated electronic structures of (Bi_1−x_Sb_x_)_2_Te_3_. (a), (b) Projected band structures of surface layer and bulk layer of pure Bi_2_Te_3_, respectively. (c), (d) Projected band structures of surface layer and bulk layer of BiSbTe_3_, respectively. (e) Total bands of BiSbTe_3_ surface layer, and the decomposed bands projected on (f) Bi, (g) Sb, and (h) Te atoms. The size of red balls corresponds to band weight, and small-size balls with tiny contributions are omitted. In other words, the size of balls indicates the intensity of states.

In order to elucidate the origin of the new surface state, first-principles calculations based on density functional theory (DFT) were performed to study the electronic structures of (Bi_1−x_Sb_x_)_2_Te_3_ with x = 0 & 0.5 (Bi_2_Te_3_ and BiSbTe_3_). The calculated band structures, as well as the layer contributions projected to both surface and bulk of Bi_2_Te_3_ and BiSbTe_3_, are displayed in Fig. 4(a–b), and Fig. 4(c–d), respectively. The TSS, in other word, DC is obtained at the Γ points for both Bi_2_Te_3_ and BiSbTe3. The positions of DP in Bi2Te3 and BiSbTe3 are about 0.11 eV and 0.05 eV below the top of bulk valence bands, respectively, showing the DC is indeed significantly pulled towards the middle of bulk band gap by doping Sb, consistent to the experimental results. Furthermore, two significant states (ST1 and ST2) were observed at 0.6 ~ 0.8 eV and 0.9 ~ 1.3 eV above Fermi energy for BiSbTe3 (Fig. 4(c)), which have larger projected intensity at surface layer. On the contrary there is only one unclear state at 0.7 ~ 0.9 eV for Bi2Te3 (Fig. 4(a)). The major states from Γ to M points are more clearly indicated by ignoring minor contribution to the bands of surface layer, as shown in Fig. 4(e).

According to the energy range of calculated surface states, the new state observed in experiment can be assigned to the ST2. In addition, there are some additional spots near the center of FFT images, shown in Fig. 2(c5–c9) with energy range of 0.5 eV–0.8 eV above EF, which can be attributed to the quasipaticles scattering in ST1. To further confirm this conclusion, we decomposed the bands of all three states to contributions from each element in the surface layer, and the results are shown in Fig. 4(f–h). We found that the DC state is a hybridized state composed by Bi-6p, Sb-5p, and Te-5p orbitals. On the other hand, the ST1 is mainly from Bi-6p and Te-5p orbitals, and the ST2 from Sb-5p orbitals. As a consequence, the ST2 can only be observed in BST sample with x ≠ 0, in perfect agreement with experiments. In addition, we found the intensity of spots in FFT images at high energy region for (Bi_1−x_Sb_x_)_2_Te_3_ become weaker by increasing the value of x (x = 0.62, and 0.87), indicating ST2 state should be more dominating for the hybrid BST sample than pure Bi_2_Te_3_ or Sb_2_Te_3_. We also compared the distributions of spin moments for the DC, ST1 and ST2 of BiSbTe_3_, as exhibited in Fig. S1. It is found that the ST2 has the similar distribution of spin with the DC from the Γ to M points.

## Discussion

Next let us discuss why the ST2 contributed by Sb atoms has a chiral spin texture. Spin degeneracy is a consequence of both time-reversal and inversion symmetry. If one of them is broken, the degeneracy can be lifted by, e.g. spin-orbit interaction. The substitution doping of Sb atoms breaks the structural inversion asymmetry in plane, leading to spin-split electronic states^24^. On the other hand, heavy atoms (Bi) are surrounded by light atoms (Sb), resulting in a strong in-plane gradient of the crystal potential in the Bi/Sb layer, which will enhance the splitting strength. The similar giant spin splitting was found in the Bi/Ag(111) surface^25^. We noted the ST2 from K-Γ-M points has nearly double parabola shape with a symmetric geometry around Γ point (Fig. 4(g)), similar to the Rashba-type spin-split bands. That may be the origination of the chiral spin texture in ST2^26^,^27^. Therefore, the spin-momentum locking can be also realized in conductance band through surface alloying effect.

The spin-momentum locking in ST2 will make the backscattering of quasiparticle in ST2 suppressed due to the protection by time-reversal symmetry. Considering the similar hexagonal warping effect, the CECs of the inner and outer spin-split subbands of ST2 are shown in Fig. 3(b) (top). Therefore, the most possible scattering vector q should be along the ΓM direction (shown as the solid arrows), which accords with experiment very well. The value of scattering vector q_H_ (dot line in Fig. 3(b)) extracted from the FFT images at high energy region should be the average of the two scattering vectors in two subbands. Similar analysis performed on QPI patterns on Bi/Ag(111) − √3 × √3 surface^26^,^27^. As a consequence, the energy dispersion E(k) of the new surface state are drew in figure 3(e–h), where q_H_ is equal to √3 k. The parabolic fitting to the dispersion give the effective mass m* about 0.56, 0.61, and 0.09 me, respectively.

The ST2 is mainly located at the first layer of BST film. When the thickness of film is only few layers even single layer, this state will be more dominated than bulk conduction states. On the other hand, this surface state is chiral, that means it may be sensitive with polarized light, which be one effective method to separate this state from bulk states. The feature of chiality in ST2 results in the prohibition the backscattering of quasiparticle due to the protection of time-reversal symmetry. So the transport properties of quasiparticle in this state will be much different with the bulk conduction states, which may be another way to separate this state from bulk states. Therefore we believe the BST films with few layers may have potential electrical or optical applications due to the existence of this chiral surface state.

In summary, we have investigated the electronic states of topological insulator BST with different x value using STM. Two robust states with chiral spin texture are found: one is Dirac surface state near the Fermi energy in samples with all x value, another state is located in conductance band in samples with x > 0 and never reported before. Combined with DFT calculations, the new spin-split states can be attributed to the substitution of Bi by Sb atoms. We think it is significant that the spin-momentum locking realize in both the topological state and the surface state stem from surface alloying in a wide energy range, which may provide more extensive applications.

## Methods

### BST sample growth and STM measurement

We prepared (Bi_1−x_Sb_x_)_2_Te_3_ thin films on Nb-doped SrTiO_3_(111) substrates with different *x* by molecular beam epitaxy (MBE), and studied the electronic structures using low-temperature scanning tunneling microscopy (STM) and scanning tunneling spectroscopy (STS). Our experiments were performed in a home-built combined low temperature STM-MBE system. The (Bi_1−x_Sb_x_)_2_Te_3_ films with thickness of 40 nm were prepared on Nb-doped single crystal SrTiO_3_(111) substrates. Details of sample preparation are described in Ref. 10. The STM images were acquired in the constant-current mode with the bias voltage applied to the tip. The dI/dV images were obtained by simultaneously recording the STM images using a lock-in technique (with 20 mV modulation at 963 Hz).

### Electronic structure calculations

The calculation model contains 15 Å of vacuum space, as well as 7 quintuple layers of 2 × 2 BST thin film. (For BiSbTe_3_, same number of Sb and Bi atoms are randomly distributed in each quintuple layer.) To better evaluate the interlayer space, the dispersion-corrected vdW-DF^28^ was employed for fully relaxation of the geometry, combined with the projector augmented wave (PAW) pseudopotential^29^,^30^ and a plane-wave basis set of 250 eV energy cutoff. The maximum force on the each relaxed atom was converged to less than 0.01 eV/Å, and the Brillouin zone was sampled by (8 × 8 × 1) k-points for both structure optimization and electronic structure calculation. All the calculations were carried out with VASP package^31^,^32^.

## Supplementary Material

Supplementary Information Supplementary Information
