# Supplementary material for: Substitution-induced spin-splitted surface states in topological insulator (Bi1−xSbx)2Te3
Source: Sci Rep. 2015 Mar 6;5:8830. doi: 10.1038/srep08830 (PMC4351528; doi:10.1038/srep08830)
Supplement: Supplementary Information [file srep08830-s1.docx]

**Supporting Materials**

**Substitution-induced spin-splitted surface states in topological insulator (Bi_1-x_Sb_x_)_2_Te_3_**

Xiaoyue He^1,2^, Hui Li^1^*, Lan Chen^1^* and Kehui Wu^1,3^

^1^ Institute of Physics, Chinese Academy of Science, Beijing 100190, China. ^2^ Key Laboratory of Standardization and Measurement for Nanotechnology, Chinese Academy of Sciences, National Center for Nanoscience and Technology, Beijing 100190, China. ^3^ Collaborative Innovation Center of Quantum Matter, Beijing 100871, China.

Correspondence and requests for materials should be addressed to: [lchen@iphy.ac.cn](mailto:lchen@iphy.ac.cn) (L. C.) & [huili8@iphy.ac.cn](mailto:huili8@iphy.ac.cn) (H. L.)


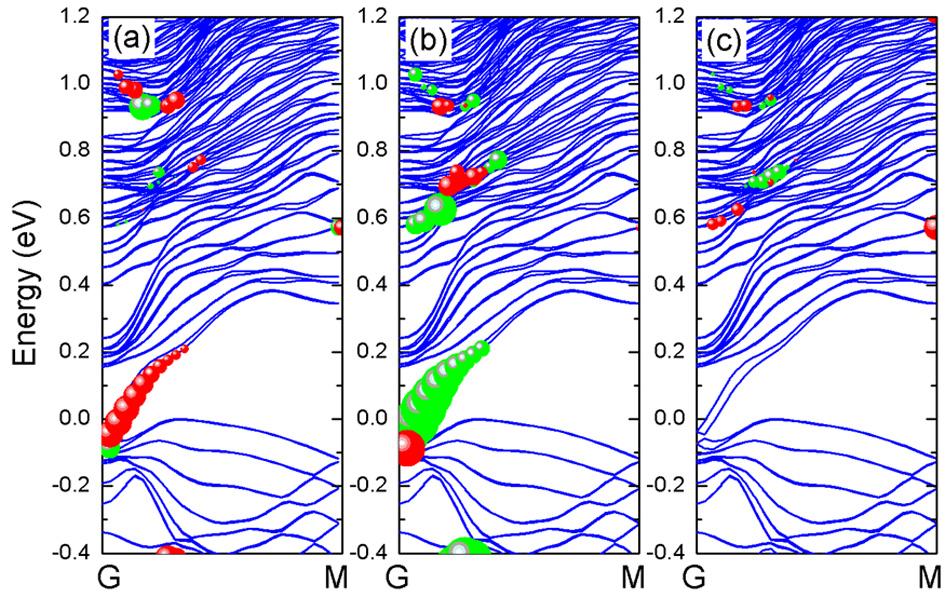


**Fig. S1.** The distributions of spin moments, (a) m_x_, (b) m_y_, and (c) m_z_, for BST surface layer. The red and green balls denote to spin moments with opposite directions, and the sizes of balls correspond to the magnitudes of moments. The small-size balls with tiny spin moments are omitted.
